# The effect of family-centered care on unplanned emergency room visits, hospital readmissions and intensive care admissions after surgery: a root cause analysis from a prospective multicenter study in the Netherlands

**DOI:** 10.1186/s13037-024-00399-8

**Published:** 2024-04-30

**Authors:** Sani Marijke Kreca, Iris Sophie Albers, Selma Clazina Wilhelmina Musters, Els Jaqueline Maria Nieveen van Dijkum, Pieter Roel Tuinman, Anne Maria Eskes, on behalf of the ARTIS consortium, Marc G.H. Besselink, Chris A. Bakker, Rosanna van Langen, Charlotte Heidsma, Marjan Ouwens, Marie-José Hendriks, Barbara L. van Leeuwen, Reggie Smith, Marthe Schreuder, Wendy Chaboyer, Hanneke van der Wal-Huisman, Johannes A. Romijn

**Affiliations:** 1 Department of Surgery, Amsterdam UMC location Vrije Universiteit Amsterdam, De Boelelaan 1117, 1081HV, Amsterdam, The Netherlands; 2 Cancer Center Amsterdam, Treatment and quality of life, Meibergdeef 9, 1105 AZ Amsterdam, the Netherlands; 3 Amsterdam UMC location Vrije Universiteit Amsterdam, Department of Anesthesiology, De Boelelaan 1117, Amsterdam, The Netherlands; 4 Amsterdam UMC location University of Amsterdam, Department of Surgery, Meibergdreef 9, 1105 AZ Amsterdam, the Netherlands; 5 Menzies Health Institute Queensland and School of Nursing and Midwifery, Griffith University, G01 2.03 Gold Coast campus Griffith University, Gold Coast, QLD 4222 Australia; 6 Department of Intensive Care Amsterdam cardiovascular Sciences Amsterdam institute for Infection and Immunity, Amsterdam UMC location Vrije Universiteit Amsterdam NL, Amsterdam, The Netherlands; 7 Intensive Care, Amsterdam UMC location Vrije Universiteit Amsterdam, De Boelelaan 1117, Amsterdam, 1081HV The Netherlands; 8 Faculty of Health, Centre of Expertise Urban Vitality, Amsterdam University of Applied Sciences, Amsterdam, The Netherlands

**Keywords:** Family caregiver, Family-centered care, Hospital, Safety, Root cause analysis, Surgery

## Abstract

**Background:**

Optimizing transitional care by practicing family-centered care might reduce unplanned events for patients who undergo major abdominal cancer surgery. However, it remains unknown whether involving family caregivers in patients’ healthcare also has negative consequences for patient safety. This study assessed the safety of family involvement in patients’ healthcare by examining the cause of unplanned events in patients who participated in a family involvement program (FIP) after major abdominal cancer surgery.

**Methods:**

This is a secondary analysis focusing on the intervention group of a prospective cohort study conducted in the Netherlands. Data were collected from April 2019 to May 2022. Participants in the intervention group were patients who engaged in a FIP. Unplanned events were analyzed, and root causes were identified using the medical version of a prevention- and recovery-information system for monitoring and analysis (PRISMA) that analyses unintended events in healthcare. Unplanned events were compared between patients who received care from family caregivers and patients who received professional at-home care after discharge. A Mann-Whitney U test was used to analyze data.

**Results:**

Of the 152 FIP participants, 68 experienced an unplanned event and were included. 112 unplanned events occurred with 145 root causes since some unplanned events had several root causes. Most root causes of unplanned events were patient-related factors (*n* = 109, 75%), such as patient characteristics and disease-related factors. No root causes due to inadequate healthcare from the family caregiver were identified. Unplanned events did not differ statistically (interquartile range 1–2) (*p* = 0.35) between patients who received care from trained family caregivers and those who received professional at-home care after discharge.

**Conclusion:**

Based on the insights from the root-cause analysis in this prospective multicenter study, it appears that unplanned emergency room visits and hospital readmissions are not related to the active involvement of family caregivers in surgical follow-up care. Moreover, surgical follow-up care by trained family caregivers during hospitalization was not associated with increased rates of unplanned adverse events. Hence, the concept of active family involvement by proficiently trained family caregivers in postoperative care appears safe and feasible for patients undergoing major abdominal surgery.

## Introduction

Adult patients who undergo major abdominal cancer surgery are at risk for significant postoperative complications such as infections, anastomotic leak or fistulas [1, 2]. Complications can lead to unplanned emergency room (ER) visits, unplanned hospital readmissions and unplanned intensive care unit (ICU) admissions [2–6]. These unplanned events can lead to worse outcomes, including increased mortality [4, 7], risk of depression, anxiety and post-intensive-care syndrome [8]. Thus, preventing these occurrences favors patient safety and well-being. The risk of complications increases in major abdominal cancer surgery patients because of the multiple transitions of care during recovery, involving various healthcare professionals during and after hospital admission [9]. Poor transitional care can lead to unplanned events [10]. Therefore, improving transitional care is of key importance to reduce complications and improve outcomes [9, 11, 12].

One way to improve transitional care is to implement transitional care interventions (TCIs) in healthcare [11, 12]. Effective TCIs focus on disease self-management education, intra- and interdisciplinary communication and co-ordination of healthcare, medication management and family engagement [11, 13, 14]. Specifically, family engagement in healthcare is a core component that appeared to be effective to improve quality of care, including enhancing patient satisfaction, improving quality of life [15, 16] and reducing hospital readmissions [11]. Its significance increases when family caregivers are more actively engaged in healthcare [11] because family caregivers provide continuity during care transitions.

To safely engage family caregivers in healthcare, conscientiously educating and training them during in-hospital healthcare is essential [17–20]. This becomes particularly crucial when patients are anticipated to require assistance with fundamental care tasks post-discharge, such as dressing, washing, mobility, and oral hygiene. In such cases, family caregivers often assume the primary caregiving role. Despite the apparent simplicity of these tasks, properly executed fundamental care holds the potential to prevent surgical complications such as pneumonia, urinary tract infections, and delirium [21–25]. Considering the potential benefits of active family involvement both during hospitalization and post-discharge, an academic hospital developed and implemented a theoretically grounded family involvement program (FIP) [26, 27]; these types of programs and other TCIs are being developed to provide healthcare professionals with tools to improve patient outcomes by refining transitional care.

Involving family in adult healthcare is practiced more often in the hospital setting, and it is relevant not only to assess its value but also to investigate its safety and potential harm. Current literature predominantly describes the positive effect on preventing adverse events when family is engaged in healthcare [18, 28]. However, in-depth research of patient safety and potential harm is currently lacking. While patients are in the hospital, healthcare professionals can address and thus prevent potential harm with the family caregiver before the family caregiver independently provides care. It remains scientifically unknown whether family caregivers can safely deliver independent, at-home care to the patient. However, based on the current evidence, we hypothesized that care provided in a home care setting by family caregivers can be safe for the patient when the family caregivers are properly trained. To assess the patients’ safety, it is necessary to research unplanned events in an in-depth manner to illuminate unintended errors caused by family caregivers that may lead to unplanned events such as ER visits, hospital readmissions and ICU admissions. Therefore, the primary aim in this study was to identify unplanned events in patients who underwent major abdominal cancer surgery and participated in the FIP, and to research their root causes. The secondary aim was to research the safety of healthcare delivered by the family caregiver at home. This was done by comparing unplanned events in patients who participated in the FIP and were cared for by the family caregiver after discharge with patients who participated in the FIP but received professional healthcare after discharge.

## Methods

### Study design

This secondary analysis of a prospective cohort study [27] involved a root cause analysis, which was performed by researching patients’ medical files using the PRISMA medical method [29]; this technique was developed to determine the causal factors of an unplanned event. Additionally, a checklist called *strengthening the reporting of observational studies in epidemiology* (STROBE) was used for reporting [30].

### Setting

The multicenter prospective cohort study was performed in the surgery departments of two hospitals in the Netherlands: the Amsterdam University Medical Center and the University Medical Center Groningen. These departments specialize in performing major abdominal cancer surgery, and healthcare providers are trained in rendering family-centered care, according to the FIP [26].

The FIP was provided to patients as part of the prospective cohort study, which commenced on April 29, 2019, and ended on May 1, 2022.

### Ethics

In the prospective cohort study, patients and their family caregivers were screened for eligibility and informed about the study at the preoperative outpatient clinic visit. If they expressed interest, they were approached by telephone by one of the researchers to receive additional information about the FIP, the possibility to participate and the required conditions while participating in the FIP. The required conditions for participation were described in our study protocol which we published separately [27]. When patient and family caregiver chose to participate, oral and written informed consent was obtained. The Medical Ethical Committee of the Amsterdam UMC (location AMC), Amsterdam, the Netherlands, granted permission to conduct this study (reference number W19-497 # 20.015).

### Intervention

Patients participated in a FIP with their family caregiver during their stay in the surgery ward; the FIP was executed post-surgery in addition to the usual postoperative care. During the FIP, family caregivers were trained by healthcare providers, including registered nurses, physical therapists and medical doctors, to execute the patient’s necessary rehabilitative healthcare. The FIP, described in detail in another article [26, 27], comprises several components: first, setting shared goals with the patient, family caregiver and nurse; second, providing information about fundamental care activities; third, task-oriented training of family caregivers to deliver fundamental care activities; fourth, establishing physical proximity by rooming-in; and fifth, training family caregivers by requiring their presence during ward rounds [18, 26, 27]. When patients were discharged from the hospital, they could opt to receive care from professionals or their trained family caregiver.

### Participants

Eligible participants for this study were selected from the intervention group of the prospective cohort study since they participated in the FIP. The patients who participated in our prospective cohort study underwent major abdominal cancer surgery. This included resections of the esophagus, stomach, colon, pancreas and liver. Patients who experienced an unplanned event were included, although patients from the control group who had an unplanned event were not included. Comparisons between the intervention and control groups were offered in the prospective cohort study [27]. One exclusion criterion was inaccessible files due to unplanned events in external hospitals.

To participate in the FIP, the following criteria were applied. First, adult patients must have scheduled major abdominal cancer surgery with an expected hospital admission of at least five days post-surgery. Second, participants had to have a family caregiver who was willing to stay during the admission and participate in the patient’s healthcare under nursing supervision [27]. Another exclusion criterion was any reason that may have prevented the family caregiver from performing safe patient care during the FIP, such as physical or mental impairments.

#### Data analysis

Patients who participated in the FIP and experienced an unplanned event were extracted from the database, which was created during the prospective cohort study. Patients’ demographic, social and clinical characteristics as well as the number and date of unplanned events were extracted from the database, as was the dichotomous variable of whether patients received professional healthcare after discharge. Unplanned events were measured until 90 days after surgery.

The primary goal in this study was to assess the safety of family engagement in post-surgical healthcare. Therefore, root causes of these unplanned events were determined to identify errors made by family caregivers which could have led to an unplanned event. Unplanned events were defined as unexpected ER visits, hospital readmissions and ICU admissions. The PRISMA medical method was used to collect, analyze and quantify information. The PRISMA medical method is a systematic method to analyze causes of unplanned events and is frequently employed in healthcare to evaluate and improve patient safety [29]. To collect information, patients’ medical records were examined and information was extracted. Results of medical and diagnostic exams and multidisciplinary reports were evaluated. After defining the main incident in the unplanned event, causal trees were created, as illustrated in the causal tree example in the Appendix in Figure 3. By continually asking why something occurred, the causes of the main event were scrutinized until the root cause was exposed. To quantify these root causes, codes were assigned according to the Eindhoven classification system [29], an algorithm to classify the type of unplanned event into main- and subcategories. The number of unplanned events per patient was collected as secondary outcome.

To reduce the risk of information bias, unplanned events in hospitals other than the university hospital were excluded from further PRISMA analyses because information concerning external unplanned events was either not accessible or could be incomplete. Excluding external unplanned events reduced the risk of information bias, although this bias remained present due to the limitation of assessing the patients’ medical records from the university hospital only. Reports of healthcare provided by professionals, such as home care nurses or general practitioners, were not consistently present in all included medical files.

To enhance reliability and objectivity, a multidisciplinary team was established, as advised in the PRISMA methodology to provide a broad spectrum of views during the evaluation of an unplanned event. Causal trees were created by two independent researchers: ISA, a medical doctor experienced in anesthesiology and intensive care and SMK, a medium-intensive-care nurse and medical master student. Both researchers followed a training course to practice the PRISMA method. The first four anonymous causal trees were tested and evaluated within the research team, who consulted an external researcher experienced with the PRISMA method to improve unity in the system of creating causal trees. After individually evaluating the unplanned events, a second joint round of analysis occurred, after which consensus was reached under supervision of a third researcher (PRT), an experienced intensivist, epidemiologist and researcher.

### Study size

All patients who participated in the FIP during the prospective cohort study, experienced an unplanned event and had complete and accessible medical records were included in this secondary analysis [31]. The sample size calculation for the inclusion of patients in the prospective cohort study is described in the study protocol [27].

#### Statistical analysis

Variables were tested for normality with the Shapiro-Wilk test and through evaluation of histograms. Descriptive statistics are presented as means ± standard deviations (SDs), medians and interquartile ranges (IQR) or numbers (percentages) when appropriate.

For the primary outcome, the PRISMA method was used [29]. Root causes were classified by the Eindhoven classification system [29], which addresses five main categories: organizational, human, technical, patient-related and unclassifiable errors. Within these categories, subcategories were defined, and codes were assigned to quantify root causes of an unplanned event [29]. In addition to the Eindhoven classification system, two subcategories were added to the unclassifiable category: unclassifiable externally, coded as *X-ex*; and unclassifiable—unrelated complication, coded as *X-nrc*. The X-ex classification was applied when the unplanned event occurred in an external hospital and the patients’ medical record was not accessible. These patients were excluded from further analysis, according to the PRISMA method. The X-nrc classification was applied when the unplanned event was a consequence of an event unrelated to the surgery or the patient’s post-surgical rehabilitation. The root causes were assigned classification codes, and then the codes were summed to determine the percentages of the total number of codes. To identify root causes which indicate an error made by the family caregiver, researchers performed the same PRISMA analysis and added another classification code to the Eindhoven classification system: unclassifiable—FIP (X-FIP). This code was also summed and presented as a percentage of the total number of codes.

For the secondary aim - to evaluate the safety of the healthcare provided by the family caregiver - the median number of unplanned events per patient was compared between patients who had and did not have professional at-home care after discharge. After testing for normality using the Shapiro-Wilk test, a Mann-Whitney U test was used. This statistical analysis was performed in SPSS, version 28.0. In this study, only complete case analyses were performed.

## Results

### Participants

The intervention group of the original prospective cohort study comprised 152 patients. Out of these 152 patients who engaged in the FIP, 68 (45%) experienced an unplanned event and were included in this root cause analysis. Among these 68 patients, a total of 116 unplanned events occurred with some patients experiencing multiple unplanned events.

### Unplanned events

**Fig. 1:**
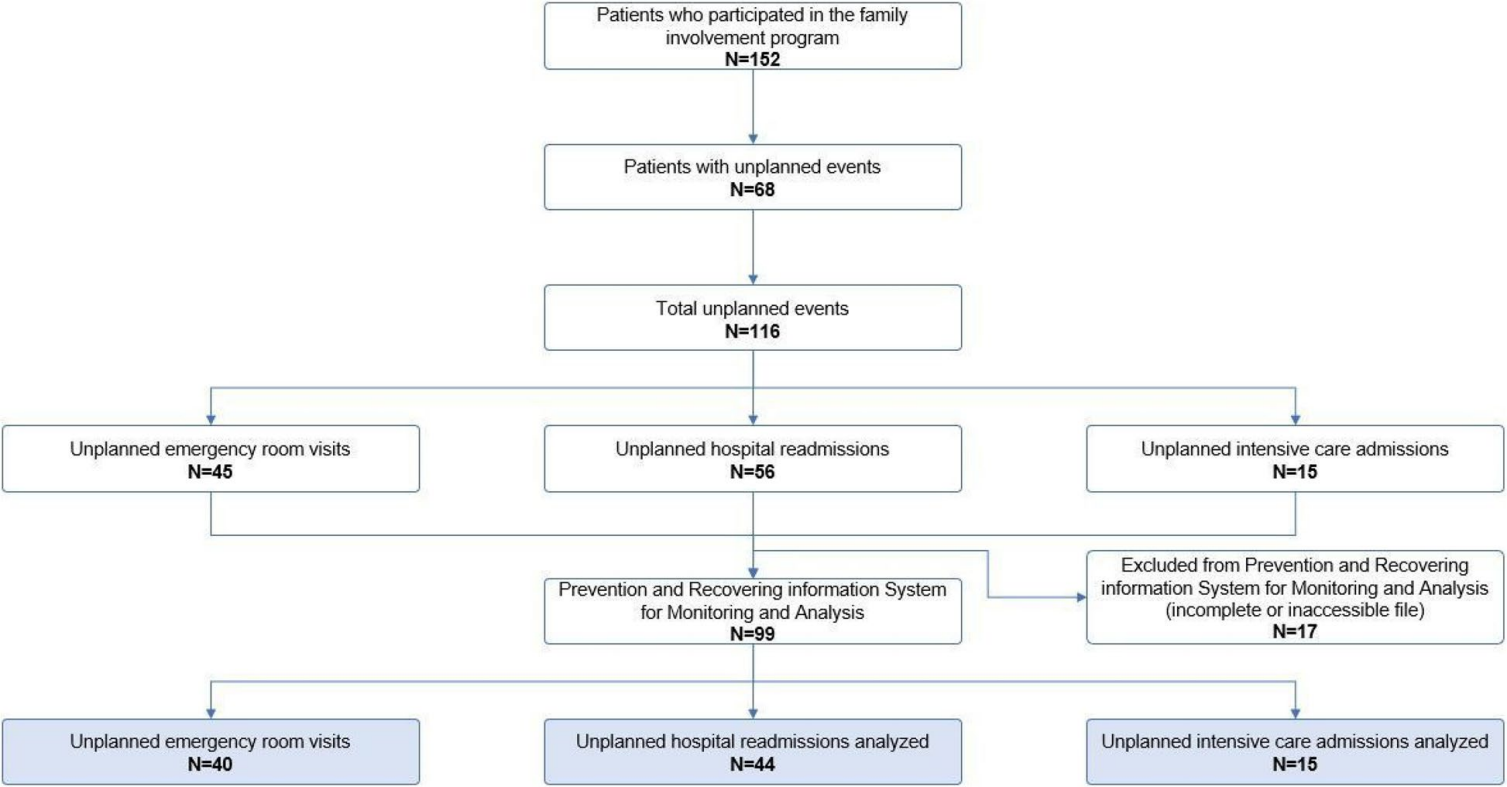
Flowchart of the enrolment of patients and the analyzed unplanned events in the root cause analysis

Out of the total 116 unplanned events, 45 were ER visits, 56 were hospital readmissions and 15 were ICU admissions. Seventeen unplanned events in 10 patients were excluded from analysis due to incomplete medical records. The enrolment of patients is presented in Fig. 1. Patients’ characteristics and clinical characteristics are described in Table 1. The mean age of the patients was 66.1 (± SD 10.1) years. Fifteen patients were female (22%). Of the family caregivers, 59 (87%) were partners and eight (12%) were children of the patient.

**Table 1:** Patient characteristics

*Patient characteristics*	
**Demographic, social and clinical characteristics**	**Total *N* = 68**
Age – mean (± SD)	66.1 (10.1)
Sex – number (%)	
-Female	15 (22)
-Male	53 (78)
American Society of Anesthesiologists (ASA) classification - number (%)	
-ASA 1	4 (5.8)
-ASA 2	33 (47.8)
-ASA 3	30 (43.5)
-ASA 4	1 (1.4)
Type of resection – number (%)	
-Esophageal	25 (37)
-Gastric	6 (9)
-Liver	6 (9)
-Pancreatic	26 (38)
-Colorectal	2 (3)
-Other	3 (4)
Polypharmacy – number (%)	
-Yes	30 (44)
-No	38 (56)
Family caregiver relationship with patient – number (%)	
-Partner	59 (87)
-Child	8 (12)
-Other	1 (1)

### Primary outcome measures

Of the 68 patients who experienced an unplanned event, 40 (59%) experienced one unplanned event and 28 (41%) experienced more than one unplanned event. Types and numbers of unplanned events are described in Table 2. Overall, 99 unplanned events were using causal trees.

**Table 2:** Descriptive statistics regarding unplanned events

Descriptive statistics regarding unplanned events	
**Descriptive data regarding unplanned events**	
Unplanned events per patient	Total (%)
-One	41 (60)
-Two	16 (24)
-More than three	11 (16)
Type of unplanned event	Total (%)
-Emergency room visit	45 (39)
-Hospital readmission	56 (48)
-Intensive care unit admission	15 (13)
-Total unplanned events	116 (100)
Unplanned events (total patients)	Median (interquartile range)
Patients who received at-home care by their trained family caregiver (*n* = 36)	36.1 (1-2)
Patients who received professional at-home care by nurses (*n* = 31)	31.1 (1-2)
Other^a^ (*n* = 1)	

^a^Patient died during the initial hospital admission after intensive care unit admission

**Fig. 2:**
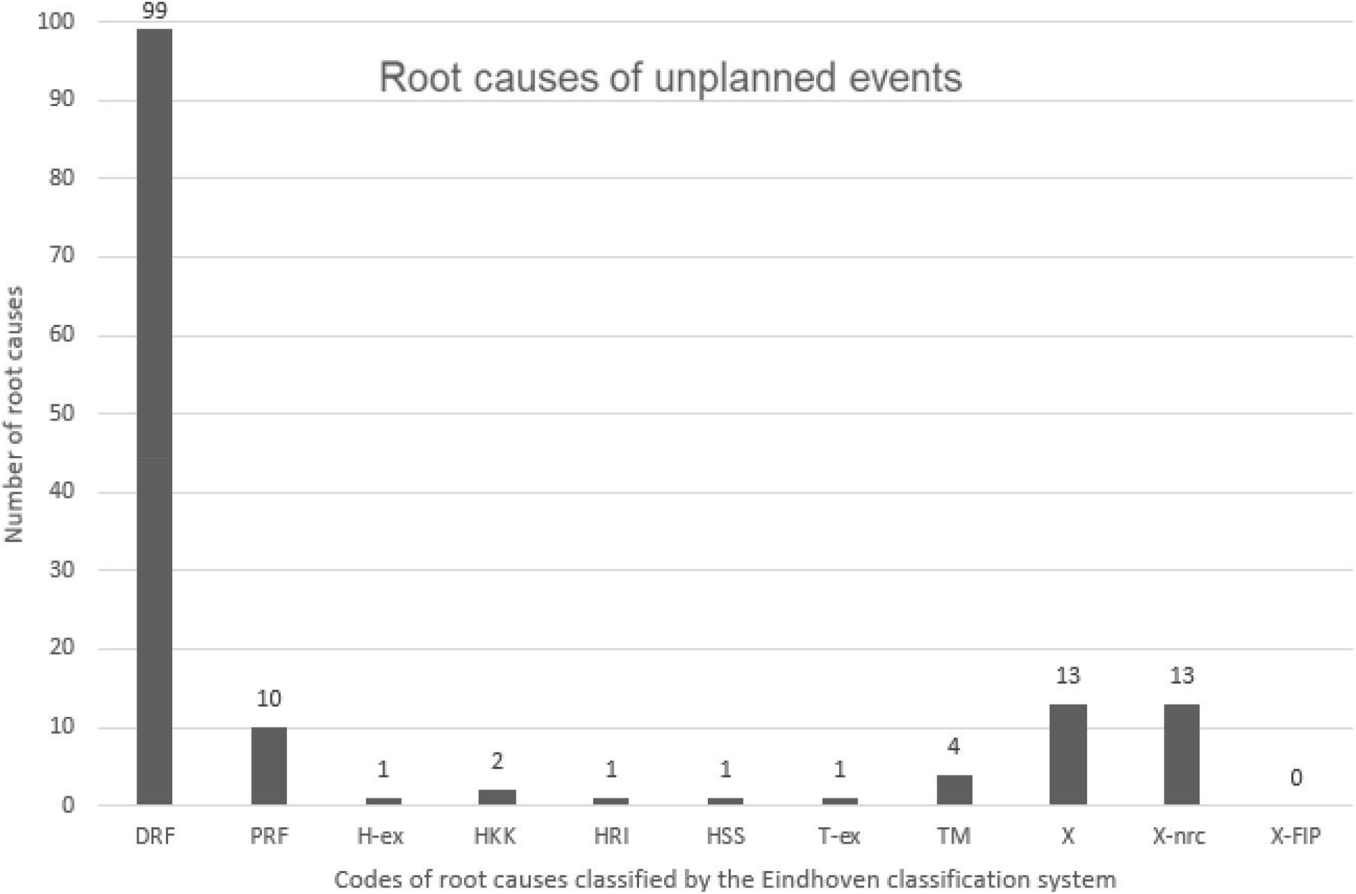
Primary outcome: root causes of unplanned events. DRF= disease related factor. PRF= patient related factor. H-ex= human external. HKK= human knowledge-based behavior. HRI= human related intervention. HSS= human skills-based. T-ex= technical external. TM= technical materials. X-nrc= unclassifiable, unrelated complication. X-FIP= unclassifiable, family involvement program

In total, 145 root causes were found, and codes were assigned according to the Eindhoven classification system [29]. Codes are defined in the Appendix in Table 3. Codes for root causes are presented in Fig. 2; most root causes were patient related (*n* = 109, 75%) and included disease- or patient-related factors, such as patient characteristics or conditions. Other root causes were related to technical errors (*n* = 5, 3%) or human errors (*n* = 5, 3%). Furthermore, unclassifiable root causes were determined (*n* = 26, 18%), of which a substantial part was unrelated to the surgery or post-surgical rehabilitation (*N* = 13, 9%). The code *X-FIP* was not seen in the data.

### Secondary outcome measures

Patients who were cared for by their family caregiver after discharge had a mean of 1.61 unplanned events. Patients who received professional care by nurses had a mean of 1.81 unplanned events. This difference was not statistically significant (*p* = 0.35).

## Discussion

In this secondary analysis of a prospective cohort study, no ER visits, hospital readmissions or ICU admissions were related to the involvement of family caregivers in post-surgical care for patients who underwent major abdominal cancer surgery. Moreover, most causes of these ER visits, hospital readmissions and ICU admission were patient related, such as disease-related complications. Furthermore, no difference in the number of unplanned events between patients who received care from a family caregiver after discharge and patients who received professional care by nurses was found, which suggests that the active involvement of family caregivers does not pose a risk to patients.

To our knowledge this is the first study to focus on the root cause analysis of unplanned events. While numerous (systematic) reviews suggest that family participation can enhance patient safety, such conclusions have predominantly relied on direct comparisons of the incidence of unplanned events, without delving into the underlying causes behind these occurrences [11, 13, 16, 32]. This additional information on patient safety can be crucial for other hospitals to implement such a program in their own setting. However, one should realize that family caregivers’ role in hospital care can be complex, given the varying legal regulations across different countries. In the context of the Netherlands, the hospital holds the ultimate legal responsibility for care. Consequently, any tasks carried out by family caregivers within the hospital must be performed under supervision, as family caregivers can make unintentional errors since they are not professionally educated to provide complex care.

Family engagement in healthcare can improve patient safety [11, 28, 33, 34]. Scientific evidence is accumulating to underscore this improvement. Nevertheless, family caregivers can make unintentional errors. Whether these errors occur and affect patient safety could not be determined from the current scientific literature, as studies regarding unintentional errors made by family caregivers are lacking.

In the current study, family caregiver engagement did not contribute to the likelihood of an unplanned event. However, family caregiver engagement also did not decrease the risk of an unplanned event which we hypothesized in the primary study (submitted data). This could be explained by the determined root causes of unplanned events. Most root causes were patient-related, which frequently meant a disease-related complication. The fact that unplanned events caused by disease-related complications occurred equally in the group who received professional homecare after discharge and the group who was taken care of by a trained family caregiver could be explained in several ways. First, the equal number of unplanned events in both groups could imply that these disease-related complications were recognized adequately by family caregivers in an early stage and that family caregivers acted upon those complications adequately by consulting medical professionals in the hospital. Second, it is possible that patients could recognize complications adequately themselves, as they were actively involved in their recovery during the FIP during the admission in the hospital when family caregivers were trained by nurses to provide care and recognize complications [27]. Improving the patients’ knowledge and self-care skills by practicing patient-centered care is also described in literature to improve the quality of care [35–37]. Another frequent root cause was categorized as unclassifiable because of a physical complication which was not related to the initial disease, such as gallstones or cardiac arrhythmias. Such unplanned events could have been caused by multiple factors including patient characteristics but were likely not influenced by those providing healthcare. Unplanned events in this study did not decrease when the family caregiver provided care after discharge; however, other benefits of family involvement like providing comfort and reassurance to the patient can be relevant to family and patients but may not affect patient safety in a direct manner [38, 39].

Safe healthcare provided by family caregivers can be beneficial on several levels. For patients, family engagement can increase the safety of care transitions and therefore decrease the risk of unplanned readmissions [11]. Preventing unplanned readmission benefits not only the patient [7], but also prevents additional healthcare costs for hospitals and healthcare systems [3]. Yet, there are further notable effects on a macro level. Home care facilities encounter difficulties with staff shortages [40, 41], which increases the workload for nurses. Not only could this lead to an enhanced risk of unintended errors [42–44] but it could also contribute to nurses’ motivation to leave the field [45]. Nursing shortages and increasing healthcare costs threaten healthcare sustainability [46], so implementing family engagement in future adult healthcare could facilitate safe solutions to providing sustainable healthcare [34, 41]. Moreover, providing family-centered care seems feasible since nurses feel more competent and positive regarding family-centered care when properly trained [47].

This study has both strengths and limitations. One strength is the generalizability of this study to patients who undergo surgery. The FIP was performed with patients who underwent complex major abdominal cancer surgery and were in need of high quality and complex postoperative care [48]. When the involvement of family in care for these patients seems to be safe, applying the FIP in less complex surgery could be a safe intervention to consider. Although this study is performed in the Netherlands which is a high-income country, its results could be meaningful and applicable to low or middle income countries as well [49–51] when professional home care nursing might be scarce due to limited resources or staff shortage [52, 53]. However, it needs to be emphasized that training of healthcare workers to provide family-centered care and training of family caregivers to participate in the patients’ healthcare requires investments of time during the hospital admission [26, 27] which is a necessity in order to provide safe care.

Another strength of this study is that patients’ medical records were analyzed completely and objectively by trained multidisciplinary medical professionals, which enabled the acquisition of diverse insights concerning unplanned events that involve different aspects of healthcare and different healthcare professionals [29, 54]. The PRISMA method itself is another strength of this study, as it provides in-depth insight into healthcare interventions on a larger scale [54], and systematic analysis of unplanned events could indicate organizational safety flaws [54]. The reliability was ensured by the individual assessment of data by qualified and PRISMA trained medical professionals. Although PRISMA is a valid method to research organizational errors in an in-depth manner, its results should be interpreted with caution. The root causes of the unplanned events could only be counted when the researchers could assess the complete medical record of the patients in the hospital. Reports of home care nurses were not accessible which might contain information about the motive of some root-causes. This is a limitation of the study. Nevertheless, causal trees were created since ER admission reports and accounts of planned hospital visits during recovery often contained detailed information about the patients’ condition after discharge. Additionally, the retrospective assessment of medical files might have led to missing data when details were not reported in the patients’ medical file.

### Clinical implications and future research

Our study enhances the existing knowledge that family participation during hospitalization can be safe by exploring the root causes of unplanned events. While initially developed for the oncological surgical population, the FIP holds potential for broader application across various contexts. Expanding its use to other surgical populations seems to be feasible, and it may also benefit other inpatient groups, as fundamental care activities extend beyond the surgical patient population.

Adaptation of instruction based on the health literacy level of patients and their caregivers is essential. In our study, we employed a combination of hands-on training and a mobile app to inform family caregivers. For those unable to use the mobile app, a paper-based booklet with identical content was provided. Moving forward, research should prioritize addressing the diverse health literacy levels of patients and their families to ensure that safety outcomes reflect the experiences of all individuals, regardless of their health literacy level.

## Conclusion

Based on the insights from the root-cause analysis in this prospective multicenter study, it appears that unplanned emergency room visits and hospital readmissions are not related to the active involvement of family caregivers in surgical follow-up care. Moreover, surgical follow-up care by trained family caregivers during hospitalization was not associated with increased rates of unplanned adverse events. Hence, the concept of active family involvement by proficiently trained family caregivers in postoperative care appears to be both safe and feasible for patients undergoing major abdominal surgery.

## Figures and Tables

**Figure:**
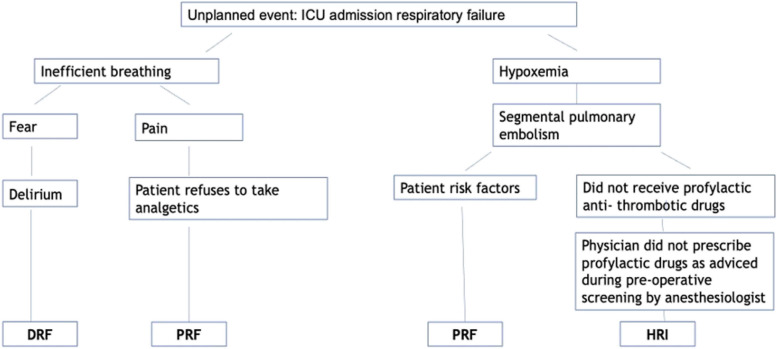
An example of a causal tree according to the Prevention and Recovery Information System for Monitoring and Analysis (PRISMA) -medical method. ICU= intensive care unit. DRF= disease related factor. PRF= patient related factor. HRI=human related factor

## Data Availability

No datasets were generated or analysed during the current study.

## Appendix

An example of a causal tree according to the PRISMA-medical method.

**Table 3:** Definitions of the Eindhoven classification model with examples

Definitions of the Eindhoven classification model with examples				
Main category	Subcategory	Code	Definition	Example
**Technical**	External	T-ex	Technical failures beyond the control of the organization.	-Vacuum wound pump was not working
	Design	TD	Failures to poor design of equipment etc.	
	Construction	TC	Correct design inappropriately constructed or placed.	
	Materials	TM	Material defects not classified under TD or TC.	-Stiches broke from the abdominal drain
**Organizational**	External	O-ex	Failures at an organizational level beyond the control and responsibility of the investigating team.	
	Transfer of knowledge	OK	Failure resulting from inadequate measures to train or supervise new or inexperienced staff.	
	Protocols	OP	Failures relating to the quality or availability of appropriate protocols.	-Not following pain treatment protocol after surgery
	Management priorities	OM	Internal management decisions which reduce focus on patient safety when faced with conflicting priorities.	-No bed available at ICU
	Culture	OC	Failure due to attitude and approach of the treating organization.	
**Human**	External	H-ex	Human failures beyond the control of the organization/department	-Impassable jejunal feeding tube as a consequence of inadequate flushing by the homecare nurse
	Knowledge based behavior	HKK	Failure of an individual to apply their knowledge to a new clinical situation	-Physician gave another order to supply fluid despite non-responsiveness of the patient in urine production and tension.
	Qualifications	HRQ	An inappropriately trained individual performing the clinical task	
	Coordination	HRC	A lack of task co-ordination within the healthcare team	
	Verification	HRV	Failure to correctly check and assess the situation before performing interventions	
	Intervention	HRI	Failure resulting from faulty task planning or performance	-No prescribed anticoagulation by the ward doctor like advised by anaesthesiologist
	Monitoring	HRM	Failure to monitor the patient’s progress or condition	
	Skills-based	HSS	Failure in performance of highly developed skills	-Luxation wound catheter
**Patient**	Patient-related	PRF	Failures related to patient characteristics or conditions, which are beyond the control of staff and influence clinical progress	-Patient did not take prescribed medication
	Disease-related	DRF	Failures related to the natural progress of disease which are beyond control of patient, its carers and staff	-Inflammation of the skin near the insertion of the abdominal drain, -Biliary pancreatitis
**X**	Unclassifiable	X	X	-Origin of blood loss objectivated by the patient was not found
		X-nrc	This root cause has no relation with the initial surgery nor rehabilitation after the initial surgery	-ER visit due to appendicitis or galstones
		X-FIP	Failures related to the healthcare delivered by the family caregiver	-Detoriation or infection of wounds as a consequence of inadequate healthcare provided by the family caregiver.

## Abbreviations

ER: Emergency room
FCC: Family-centered care
FIP: Family Involvement program
ICU: Intensive care unit
PRISMA: Prevention Recovery Information System for Monitoring and Analysis
STROBE: The Strengthening the Reporting of Observational Studies in Epidemiology
TCI: Transitional care intervention
